# Sensitive Detection of Small Particles in Fluids Using Optical Fiber Tip with Dielectrophoresis

**DOI:** 10.3390/s16030303

**Published:** 2016-02-27

**Authors:** Yi-Hsin Tai, Dao-Ming Chang, Ming-Yang Pan, Ding-Wei Huang, Pei-Kuen Wei

**Affiliations:** 1 Graduate Institute of Photonics and Optoelectronics, National Taiwan University, Taipei 10617, Taiwan; yhtai@gate.sinica.edu.tw (Y.-H.T.); dwhuang@ntu.edu.tw (D.-W.H.); 2 Department of Biochemical Science and Technology, National Taiwan University, Taipei 10617, Taiwan; b99b02014@gate.sinica.edu.tw; 3 Institute of Photonics Technologies, National Tsing-Hua University, Hsinchu 30013, Taiwan; mingyangpan@gmail.com; 4 Research Center for Applied Sciences, Academia Sinica, Taipei 11529, Taiwan; 5 Institute of Biophotonics, National Yang-Ming University, Taipei 11221, Taiwan

**Keywords:** tapered fiber tip, refractive index, dielectrophoresis, *E. coli*

## Abstract

This work presents using a tapered fiber tip coated with thin metallic film to detect small particles in water with high sensitivity. When an AC voltage applied to the *Ti*/*Al* coated fiber tip and indium tin oxide (ITO) substrate, a gradient electric field at the fiber tip induced attractive/repulsive force to suspended small particles due to the frequency-dependent dielectrophoresis (DEP) effect. Such DEP force greatly enhanced the concentration of the small particles near the tip. The increase of the local concentration also increased the scattering of surface plasmon wave near the fiber tip. Combined both DEP effect and scattering optical near-field, we show the detection limit of the concentration for 1.36 μm polystyrene beads can be down to 1 particle/mL. The detection limit of the *Escherichia coli* (*E. coli*) bacteria was 20 CFU/mL. The fiber tip sensor takes advantages of ultrasmall volume, label-free and simple detection system.

## 1. Introduction

Polluted drinking water could contain numerous of submicron/micron particles even though it looks crystal clear. Some of these small particles, such as bacteria, could cause serious illnesses to the human body such as gastrointestinal infective disease, fever, or dehydration [1,2,3]. Therefore, the fast and sensitive detection of small particles in fluids is important for monitoring the quality of drinking water. There are many methods for detecting small bacteria in water [4,5]. Most of them are based on the immunoassay techniques. However the immunoassay method needs antibody, chemicals and labels. It needs many steps and takes hours for the detections. On the other hand, optically label-free technologies are simple, real-time and cost-effective. Some methods such as Bragg grating on periodic surface structure [6,7], surface plasmon resonance on gold surface have been demonstrated for the label-free detection of bacteria or DNA [8,9,10]. In this work, we present an ultrasensitive detection method for detecting small particles in water by using a tapered optical fiber tip coated with a thin metallic film. The tapered fiber tip has a low transmission background with strong evanescent wave near the tip region due to the generation of surface plasmon wave. The scattering of the surface plasmon wave by small particles produces significant scattering near-field photons. The metal coated fiber tip also acts as a tip electrode. Combining it with a transparent electrode substrate, such as indium tin oxide (ITO) glass, a dielectrophoresis (DEP) force can be induced. By inputting a suitable alternating current (AC) frequency to the electrode, the DEP force helps concentrating the submicron/micron particles near the tip in a short time. The fiber tip can easily detect those particles because of the strong scattering of the surface plasmon wave. Optical fibers combined with DEP force for improving detection limit have been reported in previous works [11,12]. The sensing area for surface plasmon or evanescent wave and the DEP weremostly around the core of fiber. In this work, both the sensing area and DEP occurred at the fiber tip. It takes advantages of ultrasmall detection area and stronger gradient field. Only 7 μL sample and 1.5 V AC voltage were required in our experiment. We show the detection limit by the DEP concentration can be down to 1 particles/mL for 1.36 μm polystyrene beads and less than 20 CFU/mL for *Escherichia coli* (*E. coli*) bacteria. The detection time is less than 10 min.

## 2. Methodology

### 2.1. Metal Coated Optical Fiber Tip

**Figure 1:**
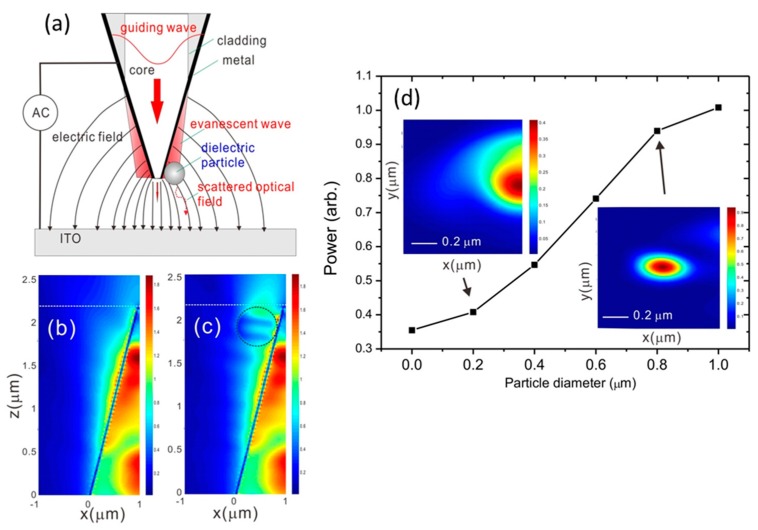
(**a**) The illustration of the detection principle; (**b**,**c**) The calculated optical fields without and with a micron sphere at the tip. The diameter of the sphere is 0.4 μm; (**d**) The optical power as a function of particle size. The inset shows the optical fields (*x*-*y* plane) at the tip end as indicated by dashed white lines in Figure 1b,c.

The sensing method is based on the scattering of surface plasmon wave near the nanometer fiber tip. Figure 1a illustrates the detection principle. The guiding wave in the core excites the surface plasmon wave near the tip. The surface plasmon wave cannot propagate to the far field as seen in the simulations of Figure 1b,c. The simulations were done by using a finite-difference time-domain method (FDTD, Fullwave, Rsoft) with a tapered core (n = 1.464), cladding (n = 1.46) and 20-nm-Al film (n = 1.23 + 7.36i). The input light wavelength was 642 nm. The Al metal is used because it has a better optical confinement effect than gold and silver [13]. For the surface plasmon wave propagating on a flat metal surface, the decay length ld (where the amplitude drops to 1/e) is determined primarily by the wavelength λ and can be express as follows:

(1) $$l_{d}=Im\left[\frac{\lambda }{2\pi }{\left(\frac{\varepsilon_{w}+\varepsilon_{Al}}{\varepsilon_{w}^{2}}\right)}^{1/2}\right]$$

where *ε_Al_* and *ε_w_* are the relative permittivities of metal and the environment. The length of the surface plasmon wave inwater is 0.417 μm. This length can be applied for measuring small particles from 0.1 μm to several micrometer. Due to the confinement of the Al film, the measured intensity of transmission light from the tip end is weaker than the intensity of surface plasmon wave. When a small particle touches the tip surface, the scattering light from the surface plasmon wave is significant due to the low transmission background. As seen in Figure 1c, the scattering intensity for 0.4 μm polystyrene sphere results in a large intensity change near the tip end. Figure 1d shows that scattering power as a function of particle diameter. The scattering power greatly increases with the particle size and is larger than the transmission background (particle size is zero). The inset shows the optical field distribution at the tip end. The scattering of surface plasmon wave by a small particle forms an optical spot near the tip.

### 2.2. Dielectrophoresis

The dielectricphoresis is a phenomenon in which a force is exerted on a dielectric particle when it is subjected to a non-uniform electric field [14,15]. All particles exhibit dielectrophoretic activity in the presence of electric fields. The strength of the force depends on the medium and particles’ dielectric properties, the particles’ shape and size, as well as on the frequency of the electric field. In principle, the force on a homogeneous sphere can be calculated by the following equation.

(2) $$F_{DEP}=2\pi r^{3}Re\left(\frac{\varepsilon_{p}-\varepsilon_{m}}{\varepsilon_{p}+2\varepsilon_{m}}\right)\nabla {\left|E_{rms}\right|}^{2}$$

where *r* is the radius of the sphere with a complex permittivity *ε_p_*.*ε_m_* is the complex permittivity in a medium. *E_rms_* is the electric field near the tip. Note that the force the force can be attractive or repulsive based on magnitude of *ε_p_*(*ω*) and *ε_m_*(*ω*). Since the permittivity is relative to AC frequency. Tuning the AC frequency could change plus (attractive) or minus (repulsive) sign in the DEP force. Gradient of the squared electric field is the main factor that affects the strength of DEP force. Input lower voltage could have stronger DEP force by a high gradient difference. There are lots of methods can increase the gradient difference,such as non-symmetrical geometric electrode [16,17], insulating nanoconstrictions [18] or nanopipettes [19] *etc*. We used non-symmetrical surface area of the sharp tip electrode to enhance the gradient differencenear the fiber tip. The ITO glass acted as the counter electrode on the other side of the fiber tip as shown in Figure 1a. The sharp fiber tip induces a high gradient of the square electric field, and finally leads to strong DEP force near the fiber tip. By tuning the input AC frequency, the sensing area of the tip could concentrate or repel spherical targets. In the meantime, the scattering of particles surrounded the tip will be increased or decreased. Compared to fiber sensor only, the DEP greatly enhances the local concentration of small particles, thus the detection limit of the electrode fiber tip is significantly improved.

### 2.3. Tapered Optical Fiber and Optical Setup

**Figure 2:**
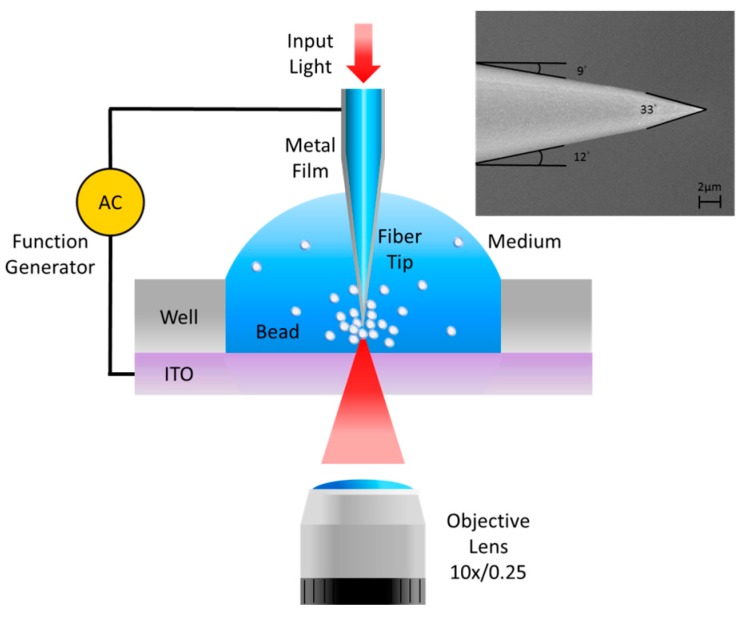
The optical setup for the measurement. A laser light passed through the chopper and coupled into the end of the fiber. The transmission light of the tip was received by an objective lens and detected by a photodetector. The function generator provided a sine function to the tip and ITO electrode. The inset shows SEM image of the fiber probe.

The fiber tip was made from a 632 nm single-mode fiber (ThorLab SM600, Newton, NJ, USA). It was formed by using a modified wet-etching method. First, the fiber jacket was removed and the bare fiber was immersed into melting polymer to form a new thick protective layer. Then it was etched by 40% (v/v) HF solution. After 60 min etching time, the protective layer was removed by acetone solution [20]. The inset in Figure 2 shows the SEM image of the fiber probe. For making a conductive tip for the DEP effect, the fiber tip was further coated with 10-nm-Ti and 35-nm-Al film by using an E-gun evaporator. The thin Ti film is necessary for increasing the adhesion of Al film on the fiber tip. Figure 2 shows the optical setup for the measurement. The distance between electrode tip and the ITO glass was maintained at a constant distance, 1 mm. A 5 mW laser light with 642 nm wavelenghth (PhoxX^®^ 642, Rodgau-Dudenhofen, Germany) passed through the chopper and coupled into the end of the fiber. The transmission light of the tip was received by an objective lens (10x/N.A. = 0.25). Then the laser intensity signal was detected by a photodetector (ThorLab DET110, Newton, NJ, USA) and read by a Lock-In (SR830 DSP, Sunnyvale, CA, USA). The function generator (SRS DS345, Sunnyvale, CA, USA) provided a sine function to the tip and ITO electrode. The AC frequency can change the direction of the DEP force on the tip. To confirm that attractive and repulsive force near the tip, we used fluorescent polystyrene beads as the particle sample. We also used the green fluorescent protein (GFP) *E. coli* for the water quality test. The excitation wavelengths for fluorescent polystyrene beads and fluorescent *E. coli* were from 460 nm to 490 nm. A CCD (pco. pixelfly) captured the emission of the fluorescence image through the dichroic mirrors. The intensity of the fluorescence signal was measured at the wavelengths ranged from 510 nm to 530 nm. The fiber tip electrode was rinsed in methanol for 5 min to avoid adhesion of polystyrene beads on the tip after each test. The deionized (DI) water and negative DEP force were used to clean the tip every time after each *E. coli* concentration test.

### 2.4. Sample Preparation

Three kinds of different size spherical particles, 3 μm polystyrene beads (FLUKA 79166, St. Louis, MO, USA), 0.1 μm fluorescence polystyrene beads (ThermoFisher F8803, Waltham, MA, USA) and 1.36 μm Polymethylmethacrylate (PMMA) beads (Bangs Laboratories Inc. PP04N, Fishers, IN, USA) were used in the experiment. The 3 μm polystyrene beads and 0.1 μm fluorescence polystyrene beads were diluted 10,000-fold by DI water. To study the detection limit of the fiber tip electrode, six different diluted ratio were made in folds of 10^5^, 10^7^, 10^9^, 10^10^, 10^11^ for 1.36 μm PMMA beads. The GFP *E. coli* were prepared by the commercial kit (pGLO™ Bacterial Transformation Kit #1660003) to obtain stable numbers of bacteria.

## 3. Results and Discussion

### 3.1. Detection of Small Dielectric Particles

**Figure 3:**
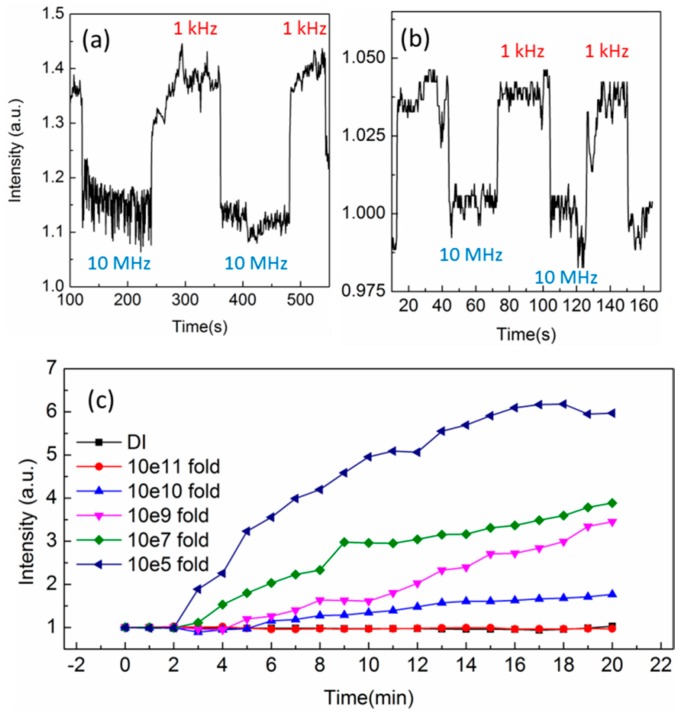
(**a**) The transmission intensity for 3 μm polystyrene beads tested by switching repeatedly from 1 kHz to 10 MHz frequency under 6 V voltage; (**b**) The transmission intensity for 0.1 μm polystyrene beads; (**c**) The transmission intensity as a function of time for different concentration. The particles were 1.36 μm PMMA beads.

We used 0.1 μm and 3 μm polystyrene beads with the same concentration to test the DEP force. A 7 μL sample droplet was dropped into a well on the ITO glass. Figure 3a shows the result of 3 μm polystyrene beads tested by switching repeatedly from 10 MHz to 1 kHz frequency under 6 V voltage [21,22]. The transmission intensity increased rapidly when changing the frequency from 1 kHz to 10 MHz. The intensity change was about 40%. Figure 3b shows the result for 0.1 μm polystyrene beads, which has the similar phenomenon as 3 μm beads. Both results confirm that there is concentration enhancement on the tip due to the DEP effect. The major difference for both different-sized spheres is the increase of intensity ratio. It was about 30% for 3 μm spheres and 3% for 0.1 μm spheres. It is noted that from Equation (2), the DEP force is proportional to r^3^ and the optical scattering cross-section is ~r^2^. The large sphere should have a strong DEP force and scattering intensity near the fiber tip. The number of particle will increase with the strength of DEP force. However, compared with the size ratio of both spheres (3/0.1), the scattering intensity difference for both spheres is only about 10 times. We attribute to the small increase of the intensity ratio for large spheres to the ultrasmall volume of effective tip region and the limited length of evanescent wave, which is about 0.4 μm in water.

The 1.36 μm PMMA bead was chosen to test the detection limit because it is shaped in symmetrical geometric form similar to *E. coli.* The original concentration was 1.4 × 10^10^ beads/mL of 1.36 μm PMMA beads and was diluted to 10^5^, 10^7^, 10^9^, 10^10^ and 10^11^ for each test. The control was the DI water. The 1.5 V, 1 kHz AC voltage was turned on after injecting the diluted solution for two min. The transmission intensity from the tip for different concentration was measured for 20 min as shown in Figure 3c. It is clear that after tenth fold dilution, the intensity difference can still be well measured. It indicates that the detection limit of 1.36 μm PMMA beads is smaller than 1 beads/mL when the DEP force is exerted on the dielectric bead.

### 3.2. Detection of E. coli

**Figure 4:**
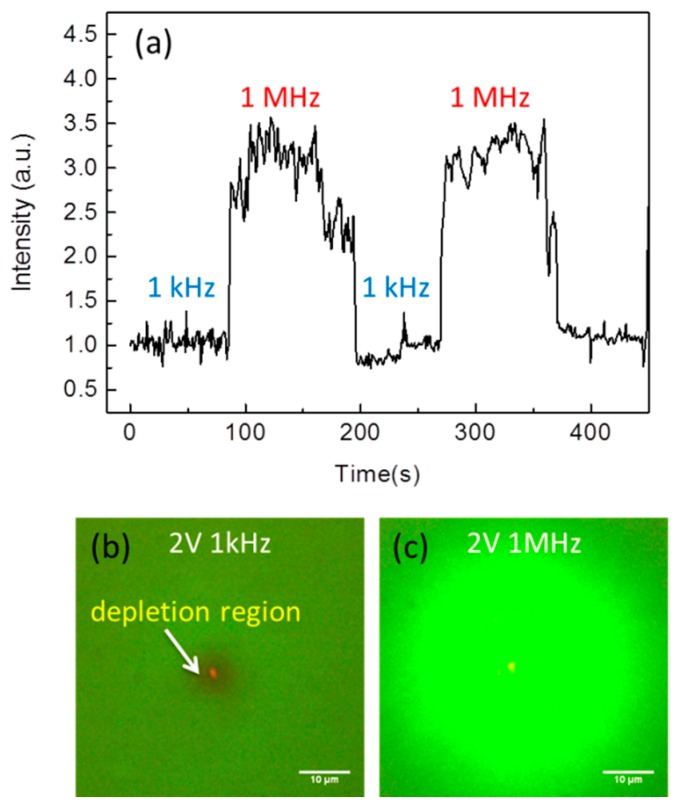
*Ti*/*Al* coating fiber was utilized to detect *E. coli* in water. (**a**) The transmission intensity for different frequency. The AC voltage was 2 V and the input frequency was switched back and forth between 1 kHz to 1 MHz. The fluorescence images of green fluorescent protein (GFP) *E. coli* under; (**b**) 1 kHz to (**c**) 1 MHz frequencies.

The *Ti*/*Al* coating fiber was utilized to detect *E. coli* in water. The AC voltage was 2 V and the input frequency was switched back and forth between 1 kHz to 1 MHz to change the sign of DEP force. Movie 1 (Video S1 in Supplementary) shows the optical images of repulsion and attraction of GFP *E. coli* at different frequency. The red optical spot indicates the transmission light from the tip end. The frequency for attracting *E. coli* was different from polystyrene and PMMA beads [23]. The transmission intensity change between attractive and repulsive DEP force was shown in Figure 4a. The intensity difference was about 3.5~4 times. Figure 4b shows the fluorescence images of GFP *E. coli*. It clearly demonstrates that negative DEP force effectively rejects *E. coli* near the tip region. Under this case, only water exists near the tip. On the other hand, Figure 4c shows the positive DEP force attracts *E. coli* near the tip. The fluorescent intensity is about four times higher than the background.

**Figure 5:**
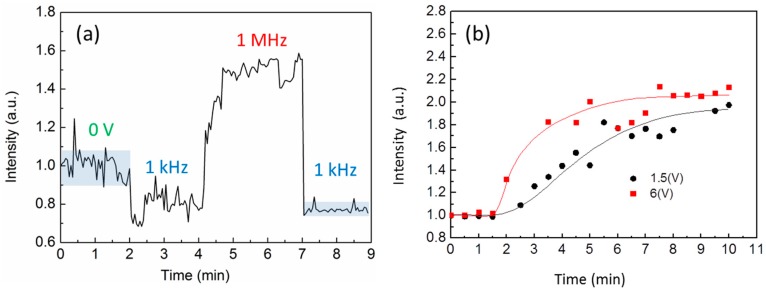
(**a**) The transmission intensity for *E. coli* solution before and after the dielectrophoresis (DEP) force. The noise was quite large at the start, voltage = 0 V. The signal was quickly decreased to the initial level when the frequency was changed to 1 kHz; (**b**) The transmission intensity as a function of time for different input voltage.

The negative DEP induces a particle depletion region. Compared to zero voltage condition, the Brownian motion of small particles causes a large random noise during the measurement. Using the negative DEP force, it can help stabilize the surrounding refractive index in *E. coli* medium as in DI water. For example, Figure 5a shows the result for high concentration of *E. coli* solution. The noise was quite large at the start. The intensity increased rapidly and gradually saturated when the frequency changed to 1 MHz. Then the signal was quickly decreased to the initial level when the frequency was changed to 1 kHz. The noise became much smaller as compared to the initial state.

**Figure 6:**
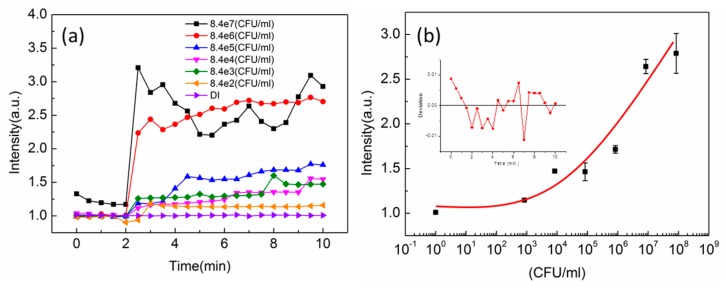
(**a**) The measured intensity as a function of time for different concentration of *E. coli*. Different concentration of *E. coli* bacteria can be clearly discriminated less than 10 min; (**b**) The measured intensity *vs.* the *E. coli* concentration at the saturation condition. The inset shows the measured noise level for low concentration of *E. coli*.

We further tested the effect of the input voltage on the response of the *E. coli* attraction. The voltage on the tip electrode was 1.5 V and 6 V respectively. The experiments were controlled at the same distance between the tip to the ITO glass and the same concentration of *E. coli*. To reduce the noise, the tip was rinsed by DI water and a negative DEP force was acted on the tip before each test. Figure 5b shows the response as a function of time for different input voltage. The voltage increases the magnitude of transmission intensity at the final stage. The major difference between different voltages is the response time. The 1.5 V DEP force had a response time 5 times longer than 6 V case. The 1.5 V took about 10 min to reach the saturation. However, in the experiment, the 1.5 V is preferred because the lower voltage could reduce the problem of the electrolysis of water. To determine the detection limit of the fiber tip sensor with DEP force, we tested different concentrations of *E. coli* with 8.4  ×  10^2^, 8.4 × 10^3^, 8.4 × 10^4^, 8.4 × 10^5^, 8.2 × 10^6^, 8.4 × 10^7^ CFU/mL and the control of DI water. The 1 kHz 1.5 V voltage was turned on for 2 min to generate repulsion force to reduce the noise level. Then the frequency was tuned to 1 MHz. Figure 6a shows the measured intensity as a function of time for different concentration of *E. coli*. *E. coli* bacteria can be clearly discriminated less than 10 min. Figure 6b shows the measured intensity and the *E. coli* concentration at the saturation condition. The fitting curve shows an exponential increase with the concentration. In the experiment, the minimum concentration of 8.4 × 10^2^ CFU/mL resulted in ~20% intensity change. The inset in Figure 6b shows the measured noise level for low concentration case. With the mean noise level of 0.5%, the detection limit of *E. coli* can achieve ~20 CFU/mL resolution.

## 4. Conclusions

The proposed *Ti/Al* coated optical fiber tip is sensitive to the environmental small particles. No pre-treatment, chemicals and labelling are necessary in the measurement. The metallic coated fiber tip and the ITO glass generates a gradient electric field on the tip. The electric field near the fiber tip results in a DEP force exerted on dielectric particles in the medium. The sign of DEP can be adjusted by AC frequency because the permittivity value of particle and medium are different from each other as in the Clausius-Mossotti factor. For negative DEP force, the surrounding contamination can be repelled out and provide a pure water environment. It helps reducing the noise level for the measurement. For positive DEP force, it greatly increases small particle numbers near the tip and thus the scattering intensity near the tip is substantially increased. With the calibration curve for each concentration, the number of the submicron/micron size bacteria can be detected with a low detection limit and short detection time. In our tests, the detection time of *E. coli* was shortened to 10 min and the detection limit was 20 CFU/mL. The detection limit of the fiber tip electrode could be further improved by optimizing metal thickness and AC voltage. The proposed method takes advantages of simple and easy measurement and can be applied for on-site diagnosis.

## Supplementary Materials

The following are available online at http://www.mdpi.com/1424-8220/16/3/303/s1, Video S1: Movie 1.
